# Utilization and accumulation of compatible solutes in *Halomonas pacifica*: a species of moderately halophilic bacteria isolated from a saline lake in South Libya

**DOI:** 10.1099/acmi.0.000359

**Published:** 2022-05-11

**Authors:** Abdolkader Abosamaha, Mike P. Williamson, D. James Gilmour

**Affiliations:** ^1^​ School of Science and Technology, Nottingham Trent University, Nottingham NG11 8NS, UK; ^2^​ Department of Molecular Biology and Biotechnology, University of Sheffield, Sheffield S10 2TN, UK

**Keywords:** halophilic bacteria, *Halomonas *bacteria, compatible solutes, extreme environments, ectoine

## Abstract

When grown in high salt concentrations, halophilic bacteria often accumulate compatible solutes, which have major applications in biotechnology because they stabilize cells and proteins. Four Gram-negative bacterial strains, belonging to the family *Halomonadaceae,* were isolated from Qaberoun and Um-Alma lakes in South Libya using high-salinity medium. The strains were identified using 16S rRNA gene sequencing as belonging to *Halomonas pacifica* (strain ABQ1)*, Halomonas venusta* (ABQ2), *Halomonas elongata* (ABU1) and *Halomonas salifodinae* (ABU2). *H. pacifica* ABQ1 is a moderate halophile (salinity range 0.05 to 2.5 M NaCl), with a broad tolerance to pH (7 to 9) and temperature (25–37 °C). Addition of the compatible solutes glycine betaine (betaine) and ectoine (1,4,5,6-tetrahydro-2-methyl-4-pyrimidine carboxylic acid) to the medium had a positive effect on growth of *H. pacifica* at 2 M NaCl. In rich LB medium, betaine was the major compatible solute accumulated, with ectoine only being accumulated at salinities in excess of 1 M NaCl. In minimal M9 medium, betaine was not produced, but increasing amounts of ectoine were synthesized with increasing salinity, and hydroxyectoine [(4S,5S)−5-hydroxy-2-methyl-1,4,5,6-tetrahydropyrimidine-4-carboxylic acid] was also synthesized when the cells were grown in very high salt. We have thus identified *H. pacifica* as a producer of ectoine and hydroxyectoine, with more being produced at higher salinities. As industrial demand for these compatible solutes continues to increase, this system has biotechnological potential.

## Introduction

Although a small percentage of the overall biosphere, hypersaline environments are found on all continents of the world. Most hypersaline aquatic ecosystems are classified as either thalassohaline (based on seawater rich in Na^+^ and Cl^−^) or athalassohaline (based on non-marine waters with high concentrations of K^+^ or Mg^2+^) [1]. Typical examples of thalassohaline environments are solar salterns, either human constructed to produce sea salt or naturally occurring extreme environments, in both cases produced by the evaporation of seawater [2]. Hypersaline alkaline lakes (soda lakes) are naturally occurring athalassohaline environments dominated by sodium carbonate where pH values commonly exceed 10 [3]. They are often formed in areas with a dry climate as a result of complex geological and biogeochemical interactions. These ecosystems provide stable, high pH habitats (usually around 9–12) because of the high buffering capacity of sodium carbonate [4].

Organisms found in saline habitats are classified as moderately halophilic if they grow optimally between 0.2 and 2.0 M NaCl, with a growth range from 0.1 to 4.5 M NaCl. Extreme halophiles show optimum growth between 3.0 and 5.0 M NaCl and have a growth range from 1.5 to 5.5 M NaCl, whereas halotolerant microorganisms grow optimally between 0 and 0.3 M NaCl with a growth range from 0 to 1 M NaCl [5]. Halophilic bacteria use two different strategies for osmoregulation when exposed to high osmolarity. Many extreme halophiles use the so-called ‘salt-in cytoplasm’ strategy, which involves accumulation of inorganic ions, mainly K^+^ and Cl^−^, in their cytoplasm to maintain osmotic balance [6–8]. However, this strategy is only used by relatively few groups of extreme halophiles and moderate halophiles, because it does not allow flexibility to colonize lower salinity environments [5].

The second type (salt-out cytoplasm) is more flexible and widespread among many microorganisms in natural environments [8, 9]. It is based on excluding salt from the cytoplasm as much as possible and accumulating low-molecular-weight organic compatible solutes to provide osmotic balance. In this type, less far-reaching adaptations of the intracellular enzymatic machinery are needed [8]. Compatible solutes are defined as small organic molecules responsible for providing osmotic balance without interfering with cell metabolism [6, 10]. Many halophilic bacteria are able to synthesize their own compatible solutes such as glycine betaine and ectoine [11]. However, halophilic microorganisms are also able to take up compatible solutes (or their precursors) from the medium, which is a far more economical way to accumulate compatible solutes [12]. In the natural environment, compatible solutes can be released into the medium by the death of organisms [13] or by a reduction in external salinity. Accumulation of compatible solutes plays an important role not only in balancing the external osmotic pressure, but also in the maintenance of protein structure and stability, and increased solubility of proteins [7].

Compatible solutes, and the organisms that produce them, have attracted considerable interest in recent years [14]. Ectoine and hydroxyectoine are of particular interest, because they have particularly powerful effects in stabilizing cells and biomolecules [15, 16]. They have applications in stabilizing cells against stress [17, 18] and are used widely in skin care [19], with sales estimated at 15000 tonnes per annum [20], generating a market of tens of millions of US dollars per year [21]. They have also been proposed to be useful for treatment of eye irritation [22], allergic rhinitis [23] and Alzheimer’s disease [24]. Hydroxyectoine appears to have a specific role in heat stress protection [25]. Of particular promise is the observation that ectoine and hydroxyectoine are more effective excipients for stabilizing antibodies than trehalose [26], which promises wide application in pharmaceutical production. There is thus considerable interest in new routes for commercial production of these compatible solutes.

The aim of our study was to characterize new species of moderately halophilic bacteria isolated from Qaberoun and Um-Alma lakes in the South Libyan Sahara. Qaberoun and Um-Alma lakes are alkaline saline lakes near Awbari City in South Libya. There have been only a few reports on their non-bacterial microbial diversity before the current work was undertaken [27]. Four Gram-negative bacterial strains, belonging to the family *Halomonadaceae,* were isolated from the lakes by subjecting the isolates to high-salinity medium, and identified using 16S rRNA gene sequencing as representing *Halomonas pacifica, Halomonas venusta*, *Halomonas elongata* and *Halomonas salifodinae *. To date, very little is known about *H. pacifica *, although two recent reports have characterized polyhydroxyalkanoate production by a strain of *H. pacifica* isolated from salt lakes near Alexandria, Egypt [28, 29]. Further characterization of the *H. pacifica* strain, isolated from Qaberoun lake, was undertaken and is described in this paper.

## Methods

### Study site and sample collection

Qaberoun and Um-Alma Lakes (Figs S1 and S2, available in the online version of this article) are located in the South Libyan Sahara (Fig. S3). The salt concentrations of the lakes are four to six times higher than seawater with a pH of around 10 [27]. Qaberoun and Um-Alma lakes form closed drainage basin lakes that represent a typical example of an extreme environment that may support a wide set of ecological niches for halophilic and alkaliphilic microorganisms.

Water samples were collected in January 2010 from different points of the surface water of Qaberoun (26.803057, 13.535278) and Um-Alma lakes (26.79995, 13.52034) using 500 ml sterile bottles. Each sample was labelled at the time and a photograph taken at each sampling site. The samples were then transferred to the laboratory of the Department of Molecular Biology and Biotechnology, University of Sheffield, UK, within 3 days. Samples were stored at 4 °C until required. Chemical analyses of water samples were carried out by Yara (Table S1).

### Growth conditions and media

Luria-Bertani (LB) medium contained the following ingredients (per litre): 5 g yeast extract (L21; Oxoid), 10 g tryptone (LP0042; Oxoid), various amounts of NaCl as detailed in the growth sections below, and the pH was adjusted to 7.8 with 1 M NaOH before autoclaving. For solid medium, 15 g of bacteriological agar No1 (LP0011; Oxoid) was also added per litre. M9 Minimal Salts medium (M-6030; Sigma) was supplemented with 1 mM MgSO_4_, 1 g NH_4_Cl l^−1^, 0.1 mM CaCl_2_ and 3 g glucose l^−1^. The pH was adjusted to between 8 and 9 using 1 M NaOH. NaCl was added to the basal M9 medium to generate the range of salinities required, using the concentrations specified below. It was sterilized by autoclaving.

### Isolation of microorganisms

Initial isolation of halophilic microorganisms was carried out as follows: portions of 0.2 ml from different dilutions (initial cultures on LB medium containing 1.5 M NaCl) were spread on plates. Plates were sealed with Parafilm, to reduce evaporation, and incubated in the dark at 30 °C. Growth was monitored daily for 3 days and any single colonies arising were streaked onto a second set of fresh LB plates containing 1.5 M NaCl using sterile loops in order to produce a pure culture of cells. Once these plates were grown, single colonies from each isolate were aseptically removed and inoculated into 5 ml of the appropriate salinity LB medium, and then incubated aerobically at 30 °C for 24 h. These cultures were transferred into 250 ml conical flasks containing 50 ml of LB medium of the appropriate salinity. The flasks were closed with sponge caps, and shaken at 250 r.p.m. at 30 °C for 24 h. The strains were maintained and routinely grown on LB and M9 minimal salts medium in both liquid and on plates at various salinities.

### Cell morphology

Cell morphology was determined by Gram staining using light microscopy at ×400 and ×1000 magnification. Cells were prepared for transmission and scanning electron microscopy (TEM and SEM) by carrying out a conventional glutaraldehyde and aqueous osmium tetroxide fixation. Electron micrographs were prepared using a Philips XL-20 scanning electron microscope (Philips) at an accelerating voltage of 20 kV.

### Molecular identification of environmental isolates

#### Genomic DNA extraction

Extraction of genomic DNA from isolated strains was carried out using the commercially available KeyPrep DNA extraction kit (Anachem) following the manufacturer’s protocols. Five 1 ml aliquots of bacterial cells from well-grown overnight cultures in LB medium (e.g. OD_600nm_ of 1.2–1.4) at a salt concentration of 0.5 M NaCl and pH 8 were transferred into 1.5 ml Eppendorf tubes and then harvested by centrifugation in a bench top centrifuge at 6000 ***g*** for 2 min at room temperature. Each pellet was washed twice in 1 ml sterile distilled water to remove residual salt, after which the standard manufacturer’s protocol was followed. Genomic DNA was transferred to 1.5 ml Eppendorf tubes and stored at −20 or −80 °C for long-term storage.

### Agarose gel electrophoresis

All DNA samples were analysed by 1% (w/v) agarose gel electrophoresis with ethidium bromide staining. Gels were visualized and photographed under UV light with the Uvitec ‘Uvidoc’ mounted camera system. A successful genomic DNA extraction was verified by resolving 2 µl of genomic DNA with 8 µl of a blue loading dye by gel electrophoresis against 2 µl GeneRuler 1 kb ladder (Fermentas International).

### 16S rRNA gene sequencing

Following extraction of genomic DNA, PCR was carried out in order to amplify the 16S rRNA gene using universal primers for bacteria, forward (5′-AGRGTTTGATCCTGGCTCAG-3′) and reverse (5′-CGGCTACCTTGTTACGACTT-3′) [30]. The PCR was performed in a final volume of 50 µl containing the following: 39 µl of sterile MilliQ water, 5 µl 10× PCR buffer, 2.5 µl of 50 mM MgCl_2_, 0.5 µl forward primer, 0.5 µl reverse primer, 1 µl of 25 mM dNTPs, 1 µl genomic DNA and 0.5 µl Taq DNA polymerase (Fermentas). Amplifications were carried out in a MyCycler thermocycler (BioRad Laboratories). The PCR cycling steps consisted of a 3 min initial pre-incubation at 94 °C followed by 30 cycles of denaturation at 94 °C for 1 min, 1 min annealing at 50 °C and 1 min elongation at 72 °C, followed by a final extension step at 72 °C for 5 min.

The PCR product was purified and cleaned up using a KeyPrep PCR purification kit following the methods stated in the protocol.

Purified PCR products (ABQ2 and ABU1) were prepared and sent for sequencing to Eurofins Genomics. The sequence was then compared to other sequences using the NCBI Blast function http://www.ncbi.nih.gov/BLAST/. For strains ABQ1 and ABU2, genomic DNA was sent to the National Collection of Industrial and Marine Bacteria (NCIMB, Aberdeen, UK) for sequencing and comparison with the MicroSeq database.

### Determination of optimum salinity

Determination of salinities for optimum growth was carried out using M9 minimal salt medium containing 3 g glucose l^−1^ at a range of salt concentrations (0, 0.05, 0.1, 0.25, 0.5, 1, 1.5, 2 and 2.5 M NaCl). M9 was used because it provides a good basal seawater-like medium to which salt can be added [31]. Five millilitres of overnight cultures, grown in M9 medium at 0.25 M NaCl and pH 7.8 to mid-exponential phase, was harvested and washed twice in sterile distilled water. The washed cells were resuspended in 3 ml sterile distilled water, and 1 ml was then inoculated into 3×250 ml flasks containing 50 ml M9 minimal medium at appropriate concentrations of NaCl. All flasks were incubated at 30 °C with shaking (250 r.p.m.), and the optical density of the cells was monitored at 600 nm after overnight growth using a Helios-γ (Unicam).

### Determination of pH range for growth

Determination of the pH range for growth was carried out in M9 minimal salt medium containing glucose (3 g l^−1^) in which the pH had been adjusted to 5.5, 6, 7, 8, 9 and 10 with either NaOH or HCl (1 M) before autoclaving and was checked afterwards to ensure the pH had not changed. One millilitre from an overnight culture grown in M9 minimal medium at 0.5 M NaCl, pH 7.8, was inoculated into 4×250 ml flasks containing M9 minimal medium at the same salinity, with pH values as described above. All flasks were incubated on a shaker at 250 r.p.m., at 30 °C. Optical density (OD_600nm_) was measured over the course of the experiment.

### Growth at different temperatures

The influence of temperature on growth was studied at 25, 30 and 37 °C. The optical density (OD_600nm_) of bacteria at each temperature was determined over the course of the experiment using M9 minimal medium containing 3 g glucose l^−1^ at 1.5 M NaCl.

### Analysis of compatible solutes using NMR

Five millitres of mid-exponential phase bacterial cultures (grown in LB and M9 minimal salt medium at salinities ranging from 0.25 to 2.5 M NaCl) was harvested and centrifuged at 3000 ***g*** for 10 min. After the supernatant was discarded, the resulting pellets were washed twice in 1 ml sterile distilled water and vortexed for 1 min at room temperature. The washed cells were then transferred into 1.5 ml Eppendorf tubes and sonicated twice for 20 s with 10 s breaks between each 20 s of sonication to allow cooling of the sample. Samples were centrifuged at 6000 ***g*** for 10 min, and the resulting supernatant was transferred into 1.5 ml Eppendorf tubes, and stored at –80 °C. After a minimum of 2 h at −80 °C, the samples were freeze-dried and stored at room temperature until required for NMR analysis. The dried samples were prepared for NMR analysis by dissolving them in 500 µl of D_2_O, adding 5 µl of 100 mM trimethyl silyl propionate (TSP; standard) using 1.5 ml Eppendorf tubes. The dissolved samples were transferred into 5 mm NMR tubes for analysis. Samples were run on a Bruker Avance-I NMR spectrometer operating at 500 MHz, at 298 K. All spectra used 90° pulses and long relaxation delays for accurate quantitation.

### Effect of compatible solutes on bacterial growth

To determine the effect of compatible solutes (proline, betaine and ectoine) on growth in highly saline media, M9 minimal medium was prepared containing 2 M NaCl, at pH 8. The compatible solutes to be examined were added to 3×49 ml aliquots of this medium to give a final concentration of 1 mM. An inoculum of 2 ml of cells grown in M9 minimal medium with 2 M NaCl was added (3×50 ml) to give an initial reading (OD_600nm_) of approximately 0.1. Three flasks were prepared without addition of compatible solutes as control. The experiment was conducted at 37 °C using an orbital shaker (250 r.p.m.), and growth was monitored by optical density measurements at 600 nm over the next 10 h.

### Growth with glycine betaine as sole source of carbon

Glycine betaine was added to 49 ml M9 medium to give a final concentration of 17 mM, at salt concentrations of 0.5 and 2 M NaCl, and pH 8. Five millitres of glucose grown cells in M9 minimal salt medium was obtained and centrifuged at 3000 ***g*** for 15 min. The pellets were washed twice in 5 ml sterile distilled water. Portions of 1 ml from washed cells were then inoculated into 2×3×250 ml sterile conical flasks containing 50 ml M9 medium with glycine betaine (17 mM) at salt concentrations of 0.5 and 2 M NaCl, and pH 8. Growth was measured based on the optical density at 600 nm over 3 days.

## Results

### Isolation and identification of microorganisms

Four strains of Gram-negative bacteria were isolated from water samples taken from Qaberoun (ABQ1, ABQ2) and Um-Alma (ABU1, ABU2) lakes. The strains were selected based on their ability to grow at a range of salinities and pH values on M9 minimal medium as well as on rich LB medium. To identify the strains, genomic DNA was extracted and for ABQ2 and ABU1, 16S rDNA was amplified by PCR and sent for sequencing. For the other two strains (ABQ1 and ABU2), genomic DNA was sent to the NCIMB for MicroSeq identification. The sequences obtained for all four strains are summarized in Table S2, which shows that all four strains belong to the genus *Halomonas* and, with similarities greater than 98%, they were assigned to the following species: *H. pacifica* (ABQ1), *H. venusta* (ABQ2), *H. elongata* (ABU1) and *H. salifodina* (ABU2). Very little has been published concerning *H. pacifica* and for this reason it was selected for further characterization.

### Cell morphology of *H. pacifica* (ABQ1)

**Fig. 1.**
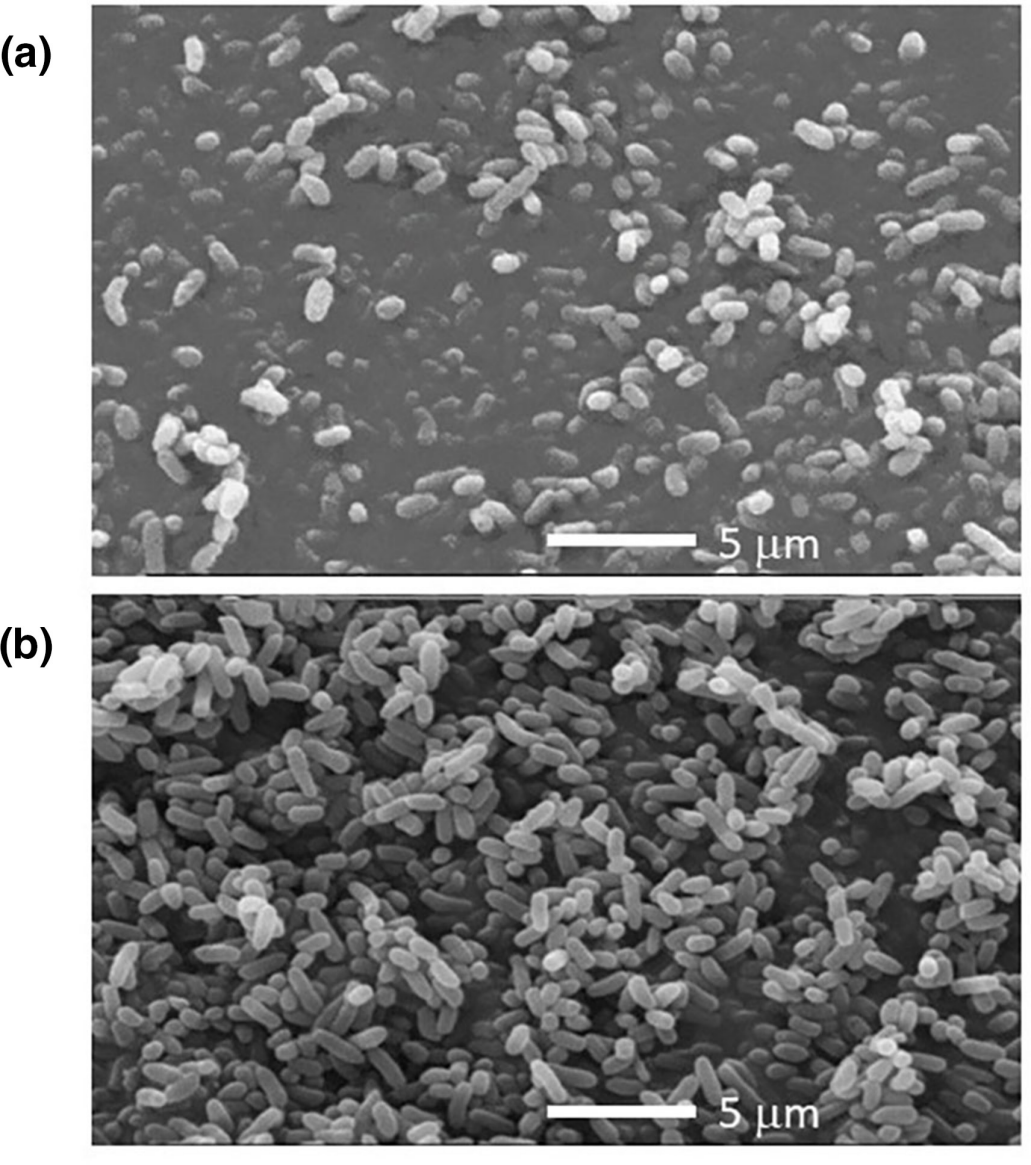
Scanning electron micrographs showing the cell morphology of *Halomonas pacifica* (ABQ1) grown in LB medium (**a**) and M9 minimal salt medium (**b**) at 0.5 M NaCl. Bars, 5 µm.

Cell morphology examined by SEM (Fig. 1) showed that for *H. pacifica* (ABQ1), growth on M9 minimal salt medium (Fig. 1b) leads to a reduction in cell size (shorter rods) when compared to cells grown on rich LB agar medium (Fig. 1a).

### Determination of optimum salinity, pH and temperature

**Fig. 2.**
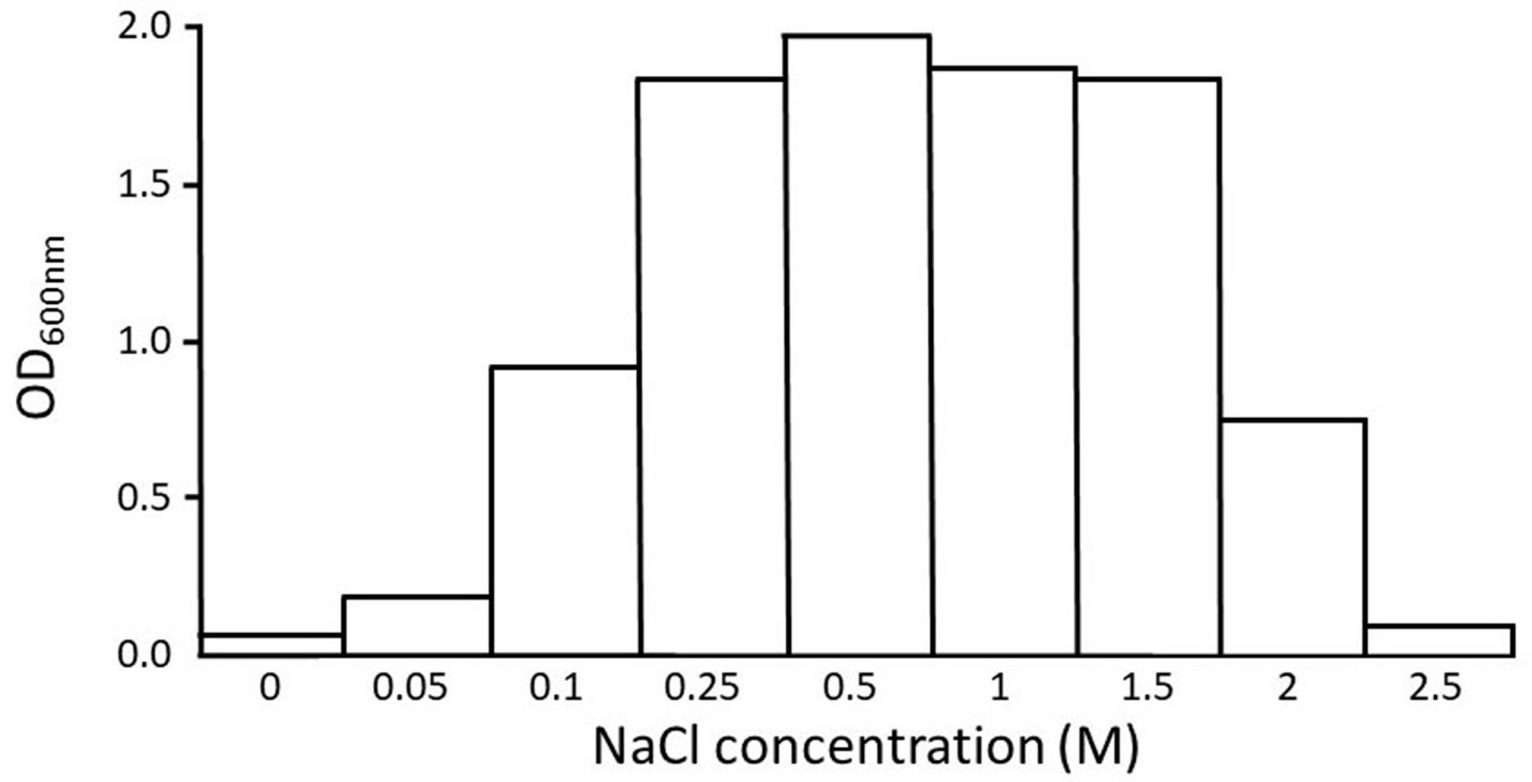
Final OD_600_ values after overnight growth of *H. pacifica* (ABQ1) in M9 minimal salt medium at pH 7.8, incubated in a 37 °C constant temperature room on an orbital shaker at 250 r.p.m. Data points are the means of triplicates. Note that the *x*-axis is not a linear scale.

Determination of the optimum salt concentration for growth of *H. pacifica* (ABQ1) was carried out at an early stage soon after isolation. Growth after overnight incubation was measured at various concentrations of NaCl, ranging from 0 to 2.5 M, in M9 minimal salt medium containing glucose, at pH 7.8. Fig. 2 shows that *H. pacifica* (ABQ1) required at least 0.05 M NaCl for growth, and can grow at up to 2 M NaCl, with optimum growth at 0.5 M.

**Fig. 3.**
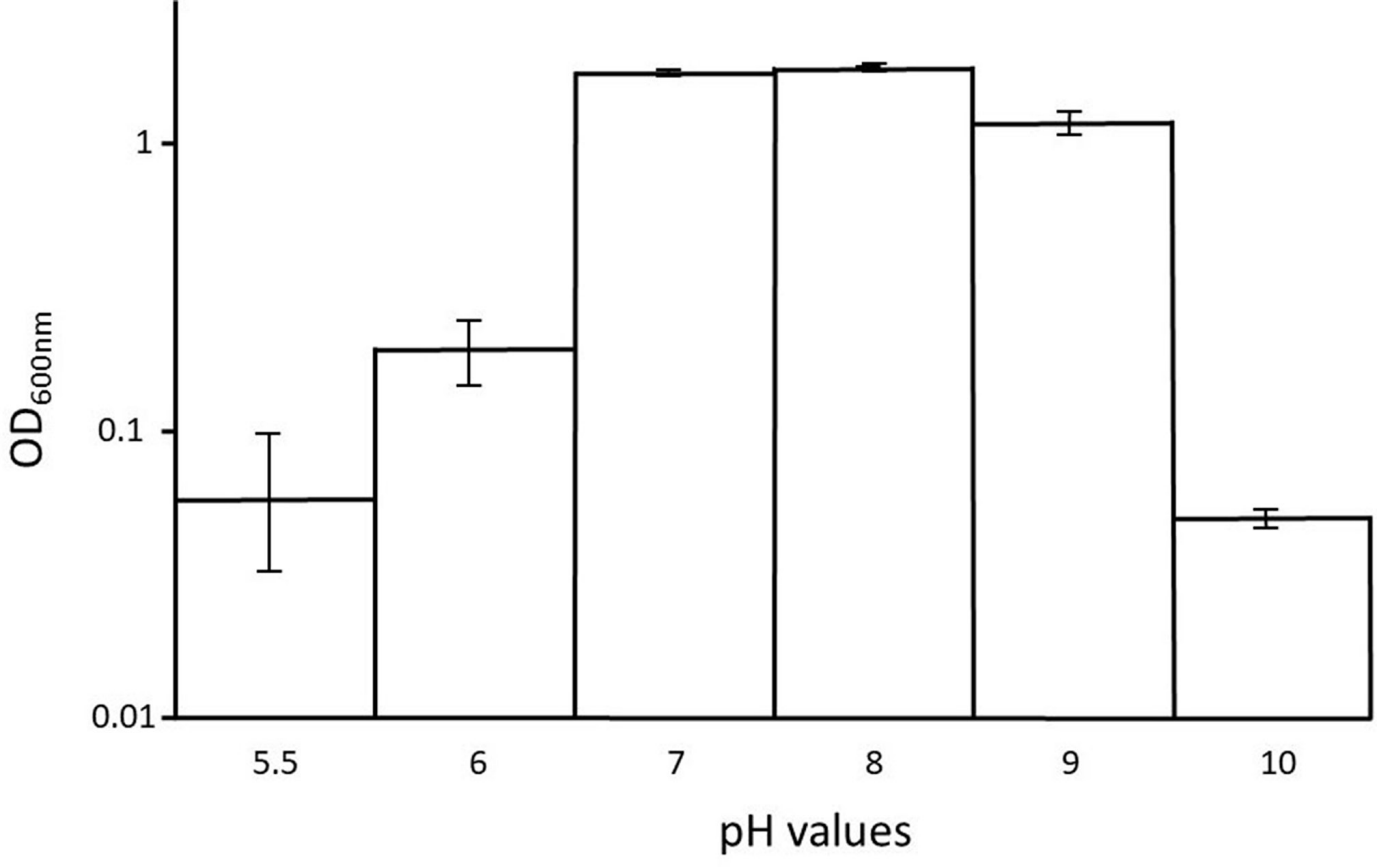
Final OD values after overnight growth of *H. pacifica* (ABQ1) in M9 minimal salt medium at 0.5 M NaCl, incubated in a 37 °C constant temperature room on an orbital shaker at 250 r.p.m. overnight. Data points are the means of triplicates and show the standard error.

Growth of overnight cultures (final OD readings) was measured for *H. pacifica* (ABQ1) to determine the optimum external pH for growth. For pH studies, the range of pH used was 5.5–10. Fig. 3 shows that *H. pacifica* (ABQ1) was able to grow best at pH 7 and 8, with good growth also seen at pH 9. Little or no growth was seen outside the range of pH 7–9.

**Fig. 4.**
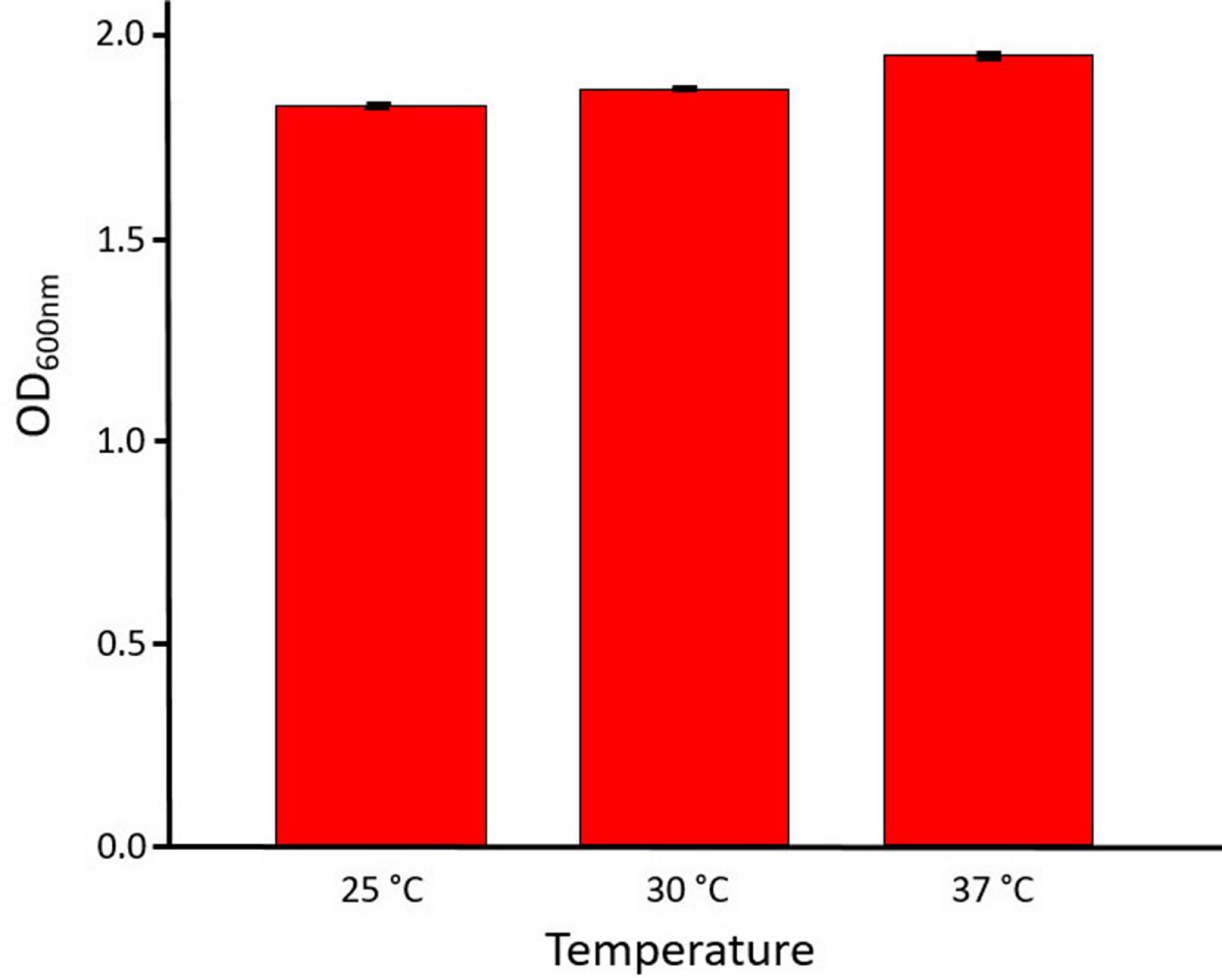
Effect of temperature on overnight growth of *H. pacifica* (ABQ1). Cells were grown in M9 minimal salt medium at 0.5 M NaCl and pH 9, incubated in a 37 °C constant temperature room on an orbital shaker at 250 r.p.m. Data are an average of three triplicates with error bars representing one standard error.

The effect of temperature on the growth rate of *H. pacifica* (ABQ1) was examined after overnight incubation and over the range 25, 30 and 37 °C. Fig. 4 shows the overnight optical density (OD_600_) for *H. pacifica* (ABQ1). The strain was capable of growth across the temperature range 25–37 °C, and optimum temperature for growth occurred at 37 °C, but in terms of final OD the values at 37 °C were only very slightly higher. The results allowed suitable upper and lower limits of temperature to be chosen for further experiments, but do not represent the temperature limits for growth.

### The role of compatible solutes on growth of *H. pacifica* (ABQ1) at high NaCl concentrations

**Fig. 5.**
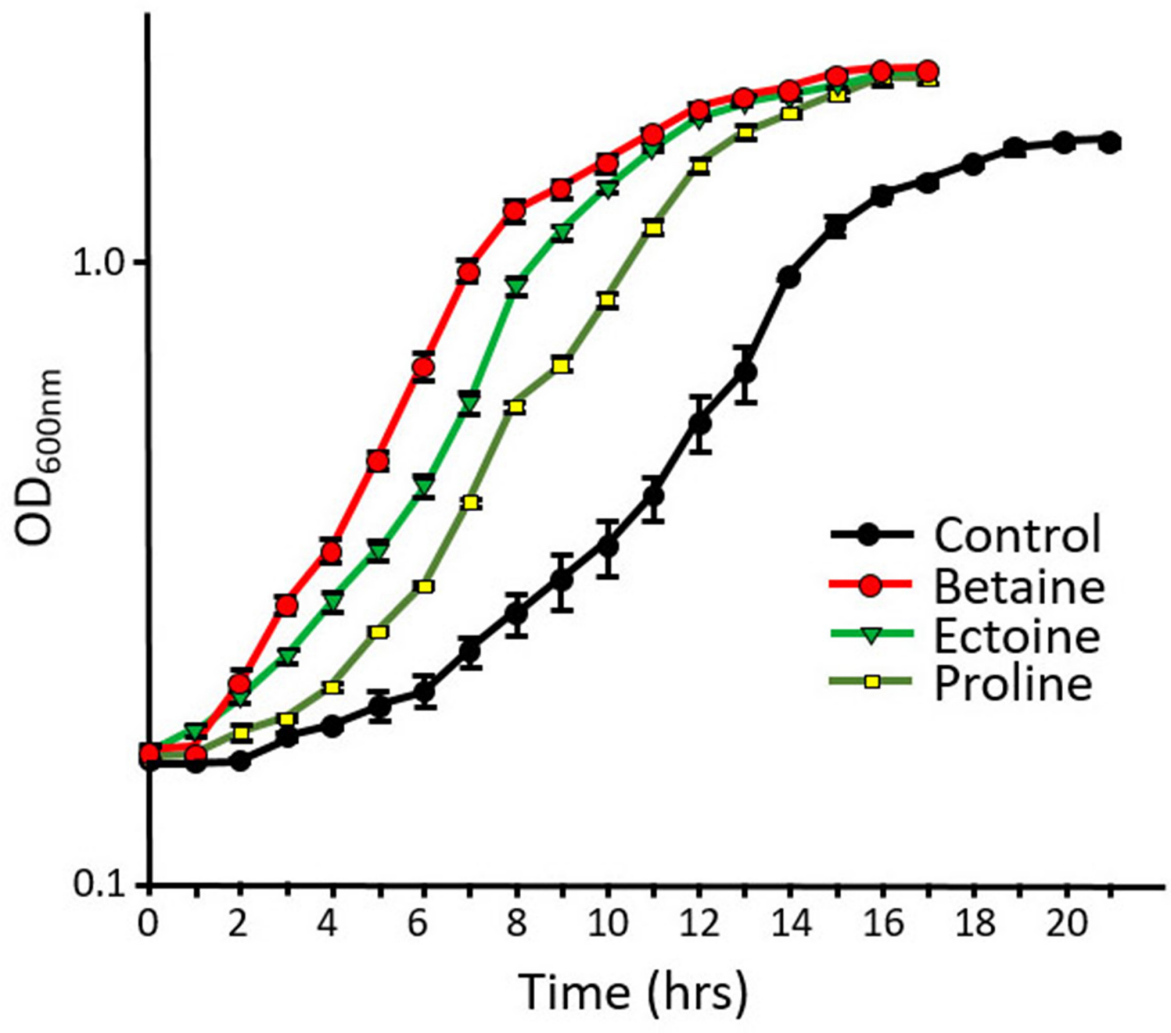
Effect of three different compatible solutes (final concentration 1 mM) on growth of *H. pacifica* (ABQ1) on M9 minimal salt medium with 2 M NaCl, at pH 8. Data presented are means of triplicate experiments with standard error. Optical density (OD_600_) was measured every hour.

**Fig. 6.**
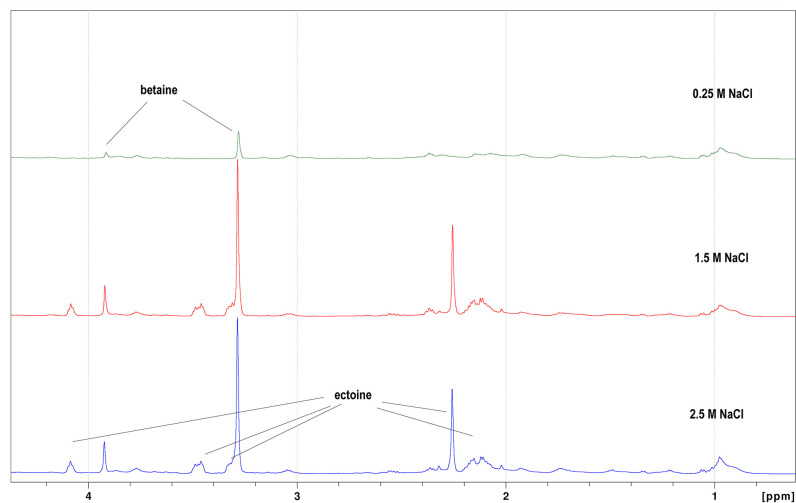
H NMR spectra of *H. pacifica* (ABQ1) cell extracts grown in LB medium at different concentrations of NaCl showing presence of betaine and ectoine.

*H. pacifica* (ABQ1) cells were grown at 2 M NaCl in M9 minimal medium in the presence of three known compatible solutes. The results presented in Fig. 5 show that all three compatible solutes have a positive effect on growth of *H. pacifica *, with betaine and ectoine having a greater effect than proline. NMR analysis was then used to determine the compatible solutes accumulated by *H. pacifica* in rich LB medium and in M9 minimal medium. Fig. 6 shows that intracellular betaine was detected at low concentrations of external NaCl in *H. pacifica* (ABQ1) grown in rich LB medium, and increased with increasing salinity up to 2.5 M NaCl. However, ectoine was only detected in the cell extracts of *H. pacifica* cells grown under high salt conditions in LB medium (1.5 M NaCl and above).

**Fig. 7.**
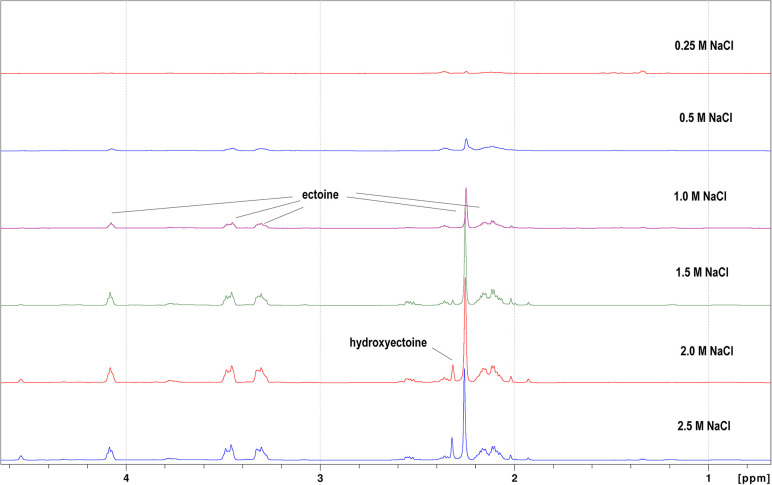
H NMR spectra of *H. pacifica* (ABQ1) grown in M9 minimal salt medium at different concentrations of NaCl showing the presence of ectoine and hydroxyectoine.

However, ectoine was the predominant compatible solute accumulated when strains were grown in M9 minimal medium, and betaine was not present (Fig. 7). There was a clear relationship between the salt concentration in M9 medium and the intracellular content of ectoine in the cells. The amount of ectoine increased significantly with increasing salinity of the medium to a maximum at 2.5 M NaCl. *H. pacifica* also accumulated hydroxyectoine when the cells were grown in M9 medium, but only at the highest salt concentrations tested, i.e. 2 and 2.5 M NaCl.

**Fig. 8.**
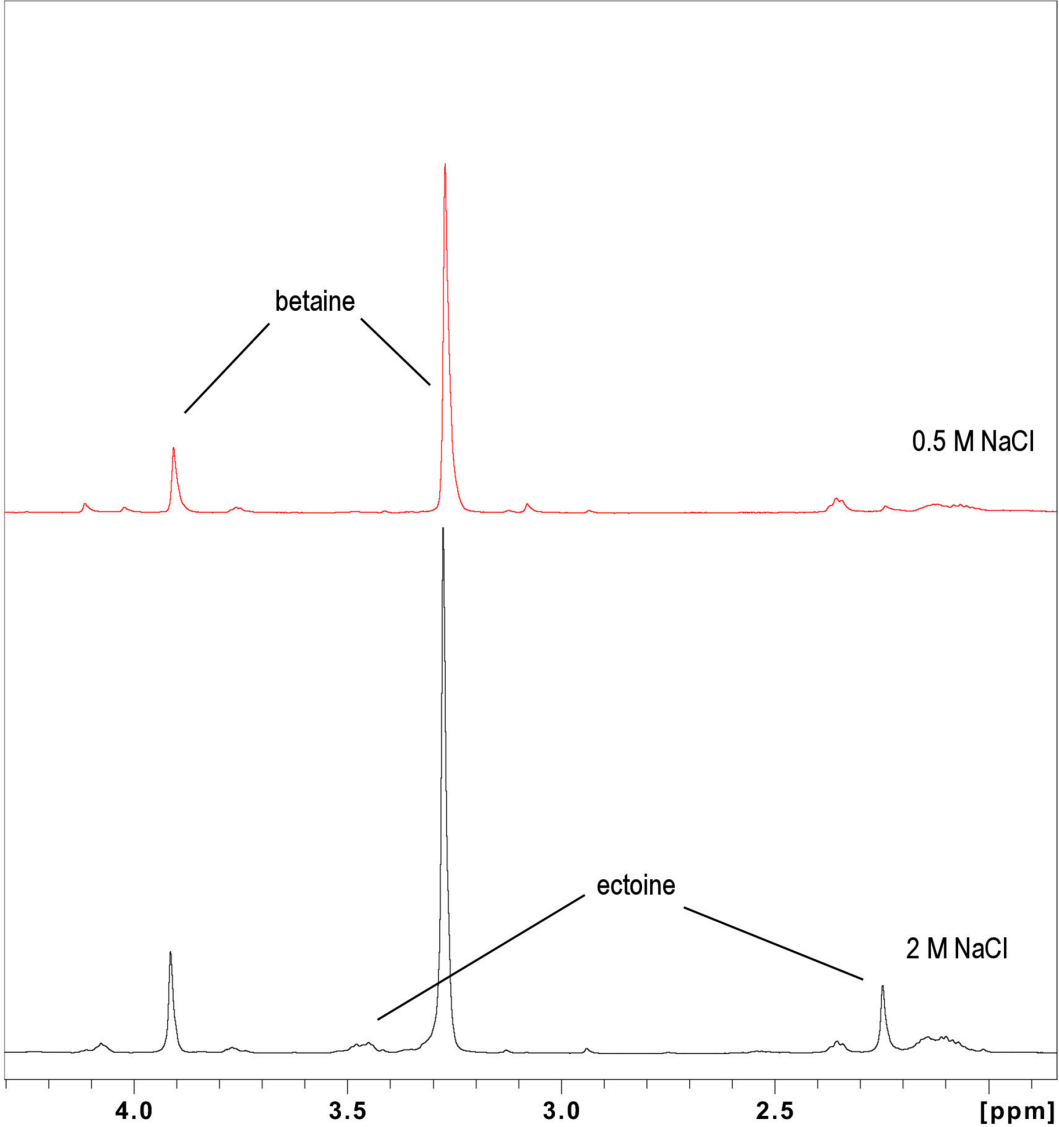
H NMR spectra of *H. pacifica* (ABQ1) grown in M9 minimal salt medium at 0.5 and 2 M NaCl, at pH 8, with 17 mM betaine as the sole carbon source, showing the presence of betaine and ectoine.

NMR spectroscopy was also used to determine which compatible solutes were being accumulated in the cytoplasm of cells grown at 0.5 and 2 M NaCl, with betaine as the sole source of carbon in the medium. Cells of *H. pacifica* (ABQ1) were pre-adapted for 2 days in M9 minimal medium containing 17 mM betaine. It is clear from the data in Fig. 8 that both betaine and ectoine accumulated when *H. pacifica* (ABQ1) was grown at 0.5 and 2 M NaCl, with betaine as the sole carbon source. The relative amounts of ectoine production compared to betaine production were 8.5 and 43% ectoine respectively at 0.5 and 2 M NaCl. Thus, *H. pacifica* (ABQ1) at low salt uses betaine almost exclusively as the compatible solute if it is available from the medium, but at higher salt it uses an increasing proportion of ectoine, when betaine is the sole source of carbon in the medium.

## Discussion

Halophilic and halotolerant microorganisms belonging to the family *Halomonadaceae* typically exist in naturally occurring saline lakes, solar salt facilities, saline soils and marine ecosystems, and haloalkaliphilic members of the *Halomonadaceae* are found in soda lakes and alkaline soils [32]. *Halomonas* is a ubiquitous and heterogeneous genus that comprises the largest number of species within the *Halomonadaceae* [33, 34]. Therefore, it is not surprising that four species of the genus *Halomonas* were isolated from Qaberoun and Um-Alma lakes.

*H. pacifica* was chosen for further study because it has not been well characterized previously. The type strain was originally isolated from a marine environment [35] and named *Alcaligenes pacificus *. It was then transferred to a newly described genus, *Deleya *, as *D. pacifica* [36]. To our knowledge, only one publication exists for *D. pacifica* [37] which characterized a restriction enzyme (*Dpa*1) found in large amounts in *D. pacifica* cells. In 1996, all species of *Deleya* were transferred to the genus *Halomonas* and the species thus became *H. pacifica* [38]. Under the name *H. pacifica *, strains have been isolated [39] and partially characterized as moderately halophilic with optimum growth at 0.5 M NaCl and a maximum salinity for growth at 3.4 M NaCl [32]. However, no detailed study of *H. pacifica* has been carried out.

The data in Fig. 2 show that *H. pacifica* (ABQ1) is a moderate halophile with optimum growth at 0.5 M NaCl in M9 minimal medium as described previously [32]. *H. pacifica* (ABQ1) showed good growth in the pH range from 7 to 9 (Fig. 3). The fact that *H. pacifica* also grows well at pH 9 with a broad temperature optimum (Fig. 4) indicates it is well adapted to grow in Qaberoun lake. A previous study reported that *H. pacifica* was able to grow over a range of temperature from 2 to 30 °C [39].

The positive effect of betaine and ectoine on the growth of halotolerant and moderately halophilic bacteria has been described in a number of previous studies [8, 40, 41]. These two compatible solutes were shown to increase the growth of *H. pacifica* when it was exposed to 2 M NaCl (Fig. 5). NMR analysis showed that betaine was the favoured compatible solute in rich LB medium (Fig. 6), presumably due to the presence of choline, which is known to act as a precursor for betaine synthesis [42]. In contrast in minimal medium, ectoine is the main compatible solute, betaine is absent and hydroxyectoine is also synthesized at the highest salinities tested (2 and 2.5 M NaCl). Therefore, the distribution of compatible solutes in rich and minimal medium is similar to other *Halomonas* species (e.g. *H. elongata *) [43, 44]. To further examine the relationship between betaine and ectoine accumulation, *H. pacifica* (ABQ1) cells were grown at 2 M NaCl with betaine as the sole source of carbon. In this situation, the cells take betaine up and use it as a compatible solute, but a portion of the betaine must also be metabolized for cell growth. The outcome was that at low salinity (0.5 M NaCl) betaine could play both roles, but at 2 M NaCl, an increasing proportion of the compatible solute accumulated was made up by ectoine, with the amount of hydroxyectoine increasing at high salt concentrations.

Ectoine and hydroxyectoine have important applications in skin care and cosmetics, and potentially in the stabilization of pharmaceutical proteins. We conclude that *H. pacifica* (ABQ1) has the potential to be a useful source organism for commercial production of ectoine and hydroxyectoine, or to provide genes for biosynthesis of these compounds for expression in other hosts such as *Escherichia coli* [45]. Hydroxyectoine has been a difficult product to isolate, because separation from ectoine is not straightforward [17]. The increased production of hydroxyectoine seen here in high-salinity medium could be a useful way to produce and extract hydroxyectoine more easily than with current biotechnological production methods.

## Supplementary Data

Supplementary material 1
