# Supplementary material for: Utilization and accumulation of compatible solutes in Halomonas pacifica: a species of moderately halophilic bacteria isolated from a saline lake in South Libya
Source: Access Microbiol. 2022 May 11;4(5):acmi000359. doi: 10.1099/acmi.0.000359 (PMC9394535; doi:10.1099/acmi.0.000359)
Supplement: Supplementary material 1 [file acmi-4-359-s001.pdf]

Supplementary material for

Utilization and accumulation of compatible solutes in *Halomonas pacifica*: a novel species of moderately halophilic bacteria isolated from a saline lake in South Libya

Abdolkader Abosamaha[1], Michael P. Williamson[2] and D. James Gilmour[2]

[1]School of Science and Technology, Nottingham Trent University, NG11 8NS, UK
[2]Department of Molecular Biology and Biotechnology, University of Sheffield, Sheffield S10 2TN, UK

Figure S1

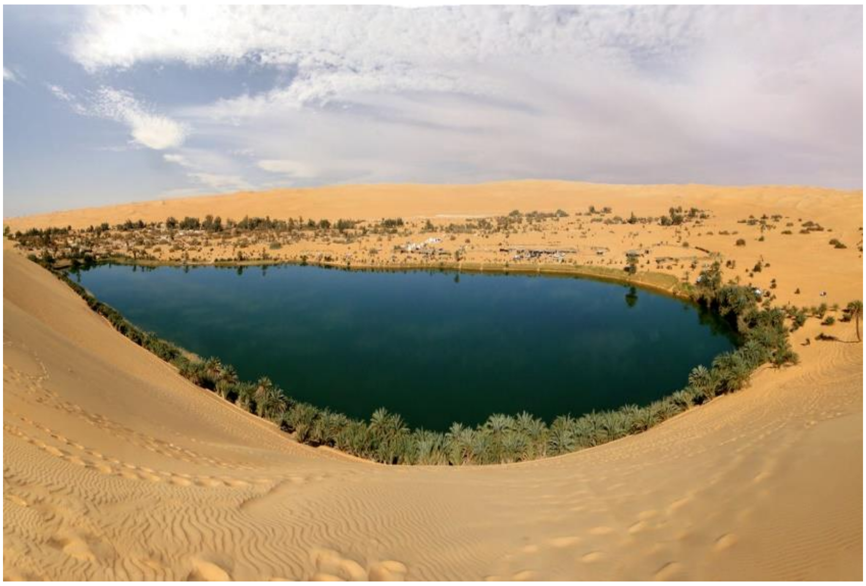

Figure S1. Qaberoun Lake. Qaberoun Lake is located in the South Libyan Sahara close to the city of Awbari, and surrounded by high sand dunes within the Awbari sand sea (Fezzan region).

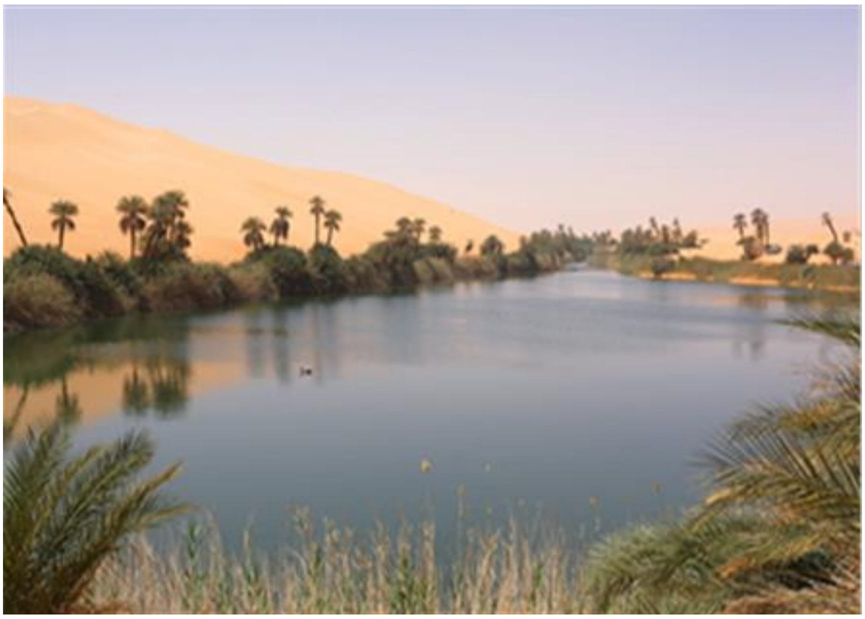

Figure S2. Um-Alma Lake. Um-Alma Lake is located close to Qaberoun Lake.

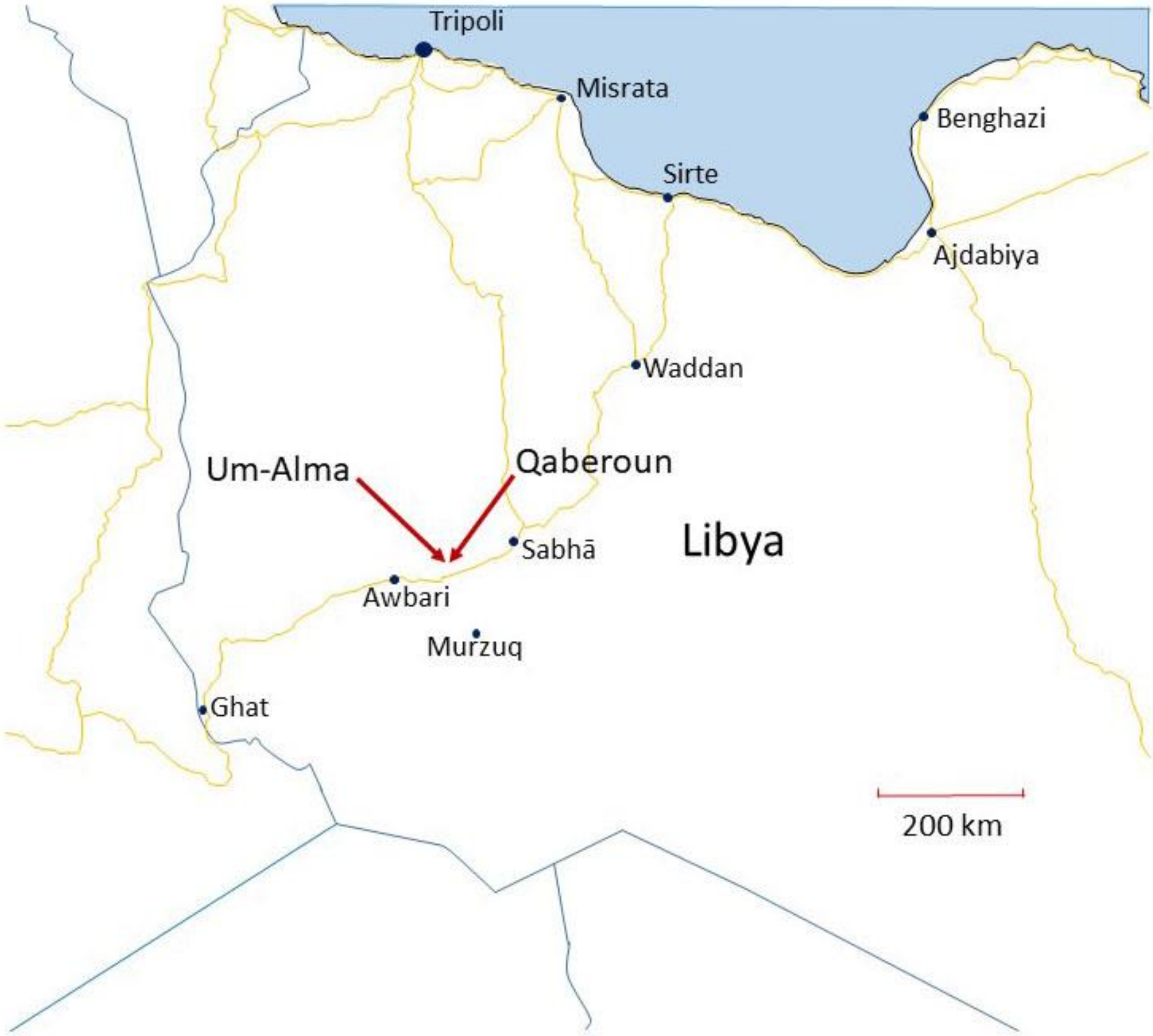

Figure S3. Map of Libya, showing the locations of Qaberoun and Um-Alma lakes. Qaberoun lake has geographic coordinates (26.803057, 13.535278), and Um-Alma lake has coordinates (26.79995, 13.52034). Adapted from Google Maps 2022 [Accessed 7 Feb 2022].

Table S1

The physical and chemical characteristics of the two lakes

**Chemical/physical parameters**	**Qaberoun lake**	**Um- Alma lake**
**pH**	9.2	9.8
**Magnesium (mg/L)**	144	20
**Manganese (mg/L)**	0.03	0.04
**Boron (mg/L)**	29.21	53.22
**Copper (mg/L)**	< 0.01	< 0.01
**Molybdenum (mg/L)**	< 0.01	0.04
**Iron (mg/L)**	0.08	0.11
**Zinc (mg/L)**	< 0.01	< 0.01
**Sulphur (mg/L)**	2883	2180
**Phosphorus (mg/L)**	< 1	2.000
**Potassium (mg/L)**	2726	2653
**Calcium (mg/L)**	13	1
**Nitrate N (mg/L)**	0.2	0.2
**EC (mmhos/cm)**	280.000	314.000
**Ammonia N (mg/L)**	0.08	0.03
**Sodium (mg/L)**	20463	21874
**Chloride (mg/L)**	79521	87906
**Bicarbonate (mg/L)**	3022	14909

Measurements were carried out by Yara Ltd. (UK).

Table S2 16S ribosomal RNA NCBI references

strain	NCBI reference
Halomonas pacifica strain NBRC 102220 (ABQ1)	NR_114047.1
Halomonas venusta strain DSM 4743 (ABQ2)	NR_042069
Halomonas elongata strain 1H9 (ABU1)	NR_074782.1
Halomonas salifodinae BC7 (ABU2)	NR_044263.1
